# Three‐Dimensional Transesophageal Echocardiographic Vena Contracta Area for Grading Mitral Regurgitation: Severity Cut‐offs From a Prospective Tunisian Cohort

**DOI:** 10.1111/echo.70609

**Published:** 2026-08-28

**Authors:** Yosra Messaoudi, Dedde El Bechir, Abdellaziz Ghadhab, Merzougui Latifa

**Affiliations:** ^1^ Faculty of Medicine of Sousse University of Sousse Sousse Tunisia; ^2^ Department of Cardiology Ibn El Jazzar Hospital Kairouan Tunisia; ^3^ Preventive Medicine Department Ibn El Jazzar Hospital Kairouan Kairouan Tunisia

**Keywords:** mitral regurgitation, rheumatic heart disease, three‐dimensional echocardiography, vena contracta area

## Abstract

**Background:**

Mitral regurgitation (MR) is among the most common valvular heart diseases, but its echocardiographic quantification remains challenging, particularly in secondary MR and in some organic causes. Three‐dimensional vena contracta area (3D VCA) allows direct planimetry of the regurgitant orifice. We aim to compare 3D VCA with two‐dimensional (2D) parameters and to derive aetiology‐specific severity cut‐offs in a Tunisian cohort.

**Methods:**

Prospective cross‐sectional study. Patients with at least moderate MR underwent transthoracic and 2D/3D transesophageal echocardiography with planimetry of the VCA. Spearman correlations between 3D VCA and 2D parameters (vena contracta width, PISA‐derived regurgitant orifice area), and diagnostic performance for severe MR (ROC, Youden index), were analyzed.

**Results:**

Ninety‐seven patients were included (mean age 61.6 ± 12.5 years; sex ratio 1.1). MR was primary in 66% (rheumatic 34%, degenerative 31%) and secondary in 34%; jets were mostly single, eccentric and holosystolic, and 62.9% had severe MR. 3D VCA correlated strongly with PISA‐derived orifice area (rho = 0.78) and with vena contracta width (rho = 0.65), particularly in organic, rheumatic, degenerative and eccentric MR. 3D VCA and 2D orifice area identified severe MR with an area under the curve of 0.93; the optimal 3D VCA cut‐off was 0.43 cm^2^ overall, 0.42 cm^2^ (rheumatic), 0.55 cm^2^ (degenerative) and 0.39 cm^2^ (secondary).

**Conclusion:**

3D VCA reliably quantifies MR, provides aetiology‐specific cut‐offs and outperforms 2D methods in complex jets. Multicenter validation is needed, particularly in rheumatic‐endemic regions.

## Graphical abstract

Three‐dimensional vena contracta area (VCA) measured by transoesophageal echocardiography enables precise accurate quantification of mitral regurgitation severity. In a Tunisian cohort, 3D VCA showed strong correlations with conventional 2D indices and excellent diagnostic performance for severe MR, supporting its use particularly in rheumatic mitral regurgitation, with an optimal threshold of 0.42 cm^2^.

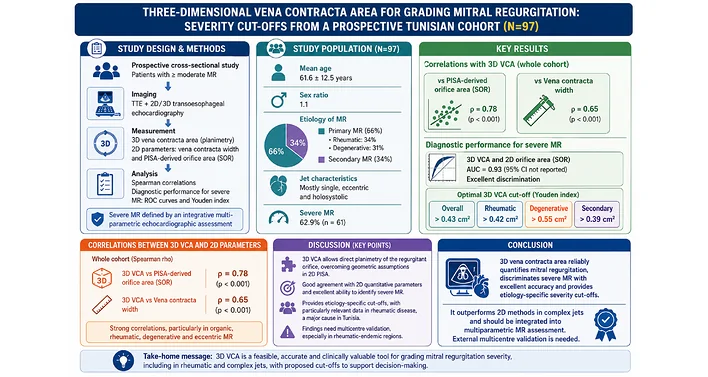

**Abbreviations**
3D VCA: three‐dimensional vena contracta area
ACC: American College of Cardiology
AHA: American Heart Association
ASE: American Society of Echocardiography
AUC: area under the curve
CI: confidence interval
CMR: cardiac magnetic resonance
EACVI: European Association of Cardiovascular Imaging
ESC: European Society of Cardiology
LVEF: left ventricular ejection fraction
MR: mitral regurgitation
NYHA: New York Heart Association
PISA: proximal isovelocity surface area
ROC: receiver operating characteristic
EROA: effective regurgitant orifice area
TEE: transesophageal echocardiography
TTE: transthoracic echocardiography
VC: vena contracta

## 1 Introduction

Mitral regurgitation (MR) is one of the most frequent valvular heart diseases in clinical practice. It affects approximately 1.7% of the general population and its prevalence rises markedly with age, exceeding 9% after the age of 75 years [1, 2]. In industrialized countries it is the second most common valvular lesion after aortic stenosis [3], whereas in low‐ and middle‐income countries moderate‐to‐severe MR affects an estimated 3%–7% of adults, with substantial regional variation reflecting the persistence of rheumatic disease [4, 5].

Echocardiography is crucial for the aetiological and severity assessment of MR and for therapeutic decision‐making. Two‐dimensional transthoracic echocardiography (2D TTE) is the first‐line modality and allows multiparametric severity grading as recommended by international guidelines [6, 7]. However, 2D approaches have well‐recognized limitations in complex situations—eccentric or multiple jets, poor acoustic windows and non‐circular regurgitant orifices—and the proximal isovelocity surface area (PISA)‐derived regurgitant orifice area relies on geometric assumptions that may not hold, especially in secondary and rheumatic MR [8, 9].

Transesophageal echocardiography (TEE), and particularly three‐dimensional TEE (3D TEE), provides superior spatial resolution and a realistic view of the mitral valve [10]. Direct planimetry of the 3D vena contracta area (3D VCA) measures the regurgitant orifice without geometric assumptions and correlates more closely with cardiac magnetic resonance than 2D Doppler methods [11, 12, 13, 14]. Current European Society of Cardiology (ESC) and American College of Cardiology/American Heart Association (ACC/AHA) guidelines recognize the value of 3D TEE for mechanism analysis, yet 3D VCA is not included among the reference criteria for severity grading [15, 16].

In Tunisia, MR is frequently encountered, but its mechanisms and aetiologies have not been systematically described, and no data exist on 3D VCA for severity assessment. We therefore conducted, in a region where rheumatic disease remains prevalent, the first Tunisian prospective study aiming to (i) describe the mechanisms and aetiologies of MR and (ii) compare the diagnostic performance of 2D (vena contracta width, PISA‐derived orifice area) and 3D (VCA) echocardiography for grading MR severity, with derivation of aetiology‐specific cut‐offs.

## 2 Methods

### 2.1 Study Design and Population

This prospective, analytical cross‐sectional study was conducted in our cardiology department over a two‐year period (May 2, 2023 to May 2, 2025), and was reported in accordance with the STROBE guidelines for observational studies. Adults (≥ 18 years) with at least moderate MR (≥grade 2) on 2D TTE who underwent a complete echocardiographic evaluation—TTE, 2D TEE and 3D TEE with direct VCA measurement—were included. Patients with congenital heart disease, previous mitral valve repair or replacement, haemodynamic instability, or contraindications to TEE were not included. Patients with insufficient image quality or non‐analyzable data were excluded.

### 2.2 Clinical Data

Cardiovascular risk factors, medical and surgical history, and New York Heart Association (NYHA) functional class were recorded on a standardized, anonymized case‐report form.

### 2.3 Echocardiography

All examinations were performed using a Vivid E9 ultrasound system (General Electric), equipped with an M5Sc transthoracic transducer (3 MHz) for transthoracic echocardiography and a 6TC multiplane transesophageal transducer for TEE. Recordings were subsequently analyzed offline using EchoPAC software.

Left ventricular (LV) volumes and ejection fraction (biplane Simpson method), LV and right ventricular dimensions, left atrial area, tricuspid annular plane systolic excursion, tricuspid S' and systolic pulmonary artery pressure were measured according to chamber‐quantification recommendations [17]. The MR mechanism was classified using the Carpentier classification [8]; aetiology was defined as primary (rheumatic, degenerative, endocarditis) or secondary (atrial or ventricular, the latter including ischaemic and dilated cardiomyopathy).

The severity of MR was classified according to the multiparametric approach recommended by the ASE and the ESC (Table 1). This approach was used as the reference standard because it does not rely on a single‐color Doppler technique is widely adopted in routine echocardiographic practice. In cases of discordance among individual parameters, MR severity was determined according to the integrated multiparametric approach. Therefore, isolated discrepancies between individual parameters did not influence patient classification.

**TABLE 1:** Multiparametric assessment of mitral regurgitation severity according to the ASE and ESC recommendations. MR severity was assigned when at least two of the three principal parameters were concordant within the same severity category, together with at least one supportive criterion.

Parameters	Moderate MR	Severe MR
**Quantitative parameters**		
Effective regurgitant orifice area (EROA), cm^2^	0.20–0.39	≥ 0.40 (≥ 0.20 in functional MR)
Regurgitant volume (mL)	30–59	≥ 60
**Semiquantitative parameters**		
Vena contracta width (mm)	3.0–6.9	≥ 7
Mitral inflow (E‐wave velocity, m/s)	—	Dominant E‐wave (>1.2 m/s)
VTI mitral/VTI LVOT	—	> 1.4
**Qualitative parameters**		
Mitral valve morphology	Normal / abnormal	Flail leaflet, large coaptation defect, sever tenting,
Color Doppler jet	—	Large central jet or large holosystolic convergence zone
Continuous‐wave Doppler signal	Dense, parabolic	Dense, triangular

**Abbreviations: CW Doppler, continuous‐wave Doppler; EROA, effective regurgitant orifice area; LA, left atrium; LVOT, left ventricular outflow tract; MR, mitral regurgitation; PISA, proximal isovelocity surface area; VTI, velocity–time integral**.

Indeed, each individual parameter has inherent limitations and may lack accuracy when used in isolation. Therefore, MR severity was assigned only when at least 2 of the following 3 quantitative parameters were concordant within the same severity category: 2D EROA, regurgitant volume, and 2D vena contracta width (VCW). In addition, at least one supportive criterion was required, including mitral inflow pattern (E‐wave velocity), continuous‐wave Doppler jet density, left atrial enlargement, or mitral valve morphological features.

### 2.4 Two‐Dimensional Quantification

The vena contracta (VC) width was measured in a zoomed parasternal long‐axis view. The surface of the regurgitant orifice (EROA; effective regurgitant orifice area) was calculated using the PISA (proximal isovelocity surface area) method [EROA = 2 πr^2^ × Va/Vmax], where r is the radius of the PISA, Va is the aliasing velocity, and Vmax is the peak velocity of the regurgitant jet. Regurgitant volume was calculated as EROA × velocity‐time integral of the regurgitant jet [18]. Severity was graded using the integrative multiparametric approach recommended by the ASE and the European Association of Cardiovascular Imaging (EACVI), which served as the reference standard; at least two quantitative or semi‐quantitative parameters had to be concordant, supported by qualitative criteria [6, 9].

### 2.5 Three‐Dimensional Vena Contracta Area

After a full‐volume color‐Doppler acquisition centered on the mitral valve (4–6 ECG‐gated cardiac cycles), multiplanar reconstruction was used to align a plane perpendicular to the jet at the level of maximal constriction, and the regurgitant orifice was traced manually to obtain the 3D VCA (cm^2^). In case of multiple jets, each orifice was measured and the areas were summed; in patients with atrial fibrillation, values were averaged over 3–5 beats. Multiparametric grading and 3D VCA measurement were performed independently and interpreted blinded to each other.

### 2.6 Statistical Analysis

Analyses used SPSS v21. Quantitative variables are expressed as mean ± standard deviation and categorical variables as counts and percentages. Correlations between continuous variables were assessed using Spearman test (rho). Diagnostic performance for severe MR was assessed by receiver operating characteristic (ROC) analysis; the optimal cut‐off was defined as the value maximizing the Youden index, and results are reported as area under the curve (AUC) with 95% confidence interval (CI), sensitivity, specificity and cut‐off. A two‐sided *p* < 0.05 was considered statistically significant.

### 2.7 Ethics and Use of Artificial Intelligence

The study was conducted in accordance with the Declaration of Helsinki and was approved by our institution ethics committee. As the investigations were performed as part of routine clinical care, patients were informed of the study and gave written consent to use their anonymized data. Generative artificial‐intelligence tools were used to assist with language editing and formatting; all content was reviewed and validated by the authors, who take full responsibility for it.

## 3 Results

### 3.1 Study Population

**FIGURE 1:**
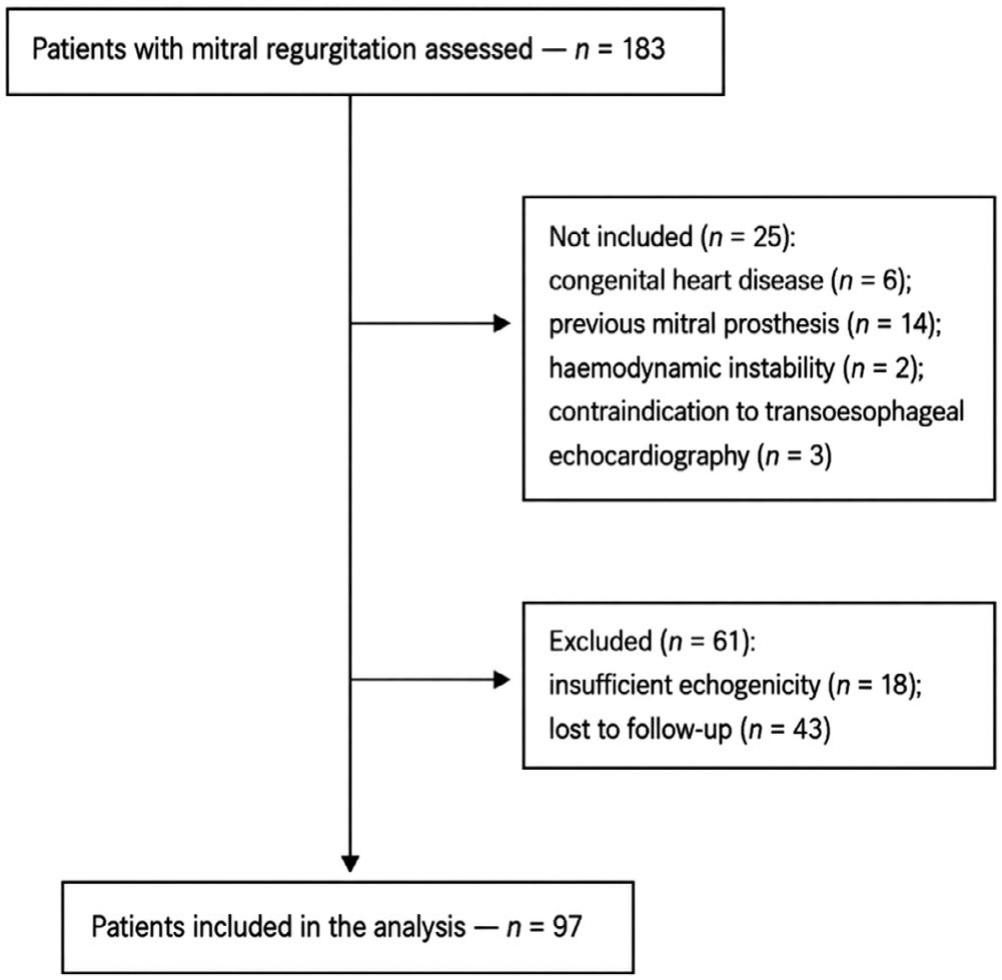
Study Flow chart. Among 183 patients with mitral regurgitation who were assessed, 97 patients were included in the final analysis.

Ninety‐seven patients met the inclusion criteria (Figure 1).

The mean age was 61.6 ± 12.5 years (range 20–88) with a balanced sex distribution (sex ratio 1.1). Hypertension was present in 47.4%, diabetes in 33.0% and atrial fibrillation in 55.7%; most patients were in NYHA class III (45.4%) or II (32.0%). Baseline characteristics are shown in Table 2.

**TABLE 2:** Baseline clinical characteristics of the study population (*N* = 97). Age is presented as mean standard deviation (range). All other categorical variables are presented as *n* (%).

Variable	Value
Age, years (mean ± SD)	61.6 ± 12.5 (20–88)
Male sex	51 (52.6)
Hypertension	46 (47.4)
Diabetes mellitus	32 (33.0)
Dyslipidaemia	35 (36.1)
Current or former smoking	38 (39.2)
Chronic kidney disease	9 (9.3)
Obesity	3 (3.2)
Atrial fibrillation	54 (55.7)
Coronary artery disease	27 (27.8)
Associated valve disease	33 (34.0)
NYHA class I / II / III / IV (*n*, %)	10 (10.3) / 31 (32.0) / 44 (45.4) / 12 (12.4)

*Note*: Values are *n* (%) unless otherwise stated.

Abbreviations: NYHA, New York Heart Association; SD, standard deviation.

### 3.2 Mechanisms and Aetiologies

By the Carpentier classification, restrictive systolic‐diastolic leaflet motion (type IIIa) predominated (39.2%), followed by prolapse (type IIa, 23.7%); annular dilatation (type Ia) and systolic restriction (type IIIb) each accounted for 16.5%. MR was primary in 66% and secondary in 34%; among secondary MR, the ventricular phenotype predominated (78.8%). The leading primary aetiologies were rheumatic (*n* = 33, 34%) and degenerative (*n* = 30, 31%), and ischaemic MR accounted for 20.6%. Regurgitant jets were mostly single (91.8%), eccentric (77.3%) and holosystolic (84.5%). Aetiologies, mechanisms and jet characteristics are detailed in Table 3.

**TABLE 3:** Aetiologies, carpentier mechanisms, and regurgitant‐jet characteristics of mitral regurgitation in the study population (*N* = 97). Values are presented as *n* (%). Percentages were calculated using the entire study population as the denominator.

Characteristic	*n* (%)
Mechanism (Carpentier)	
Type Ia—annular dilatation	16 (16.5)
Type Ib—perforation	1 (1.0)
Type IIa—prolapse	23 (23.7)
Type IIb—flail leaflet	3 (3.0)
Type IIIa—systolo‐diastolic restriction	38 (39.2)
Type IIIb—systolic restriction	16 (16.5)
**Aetiology**	
**Primary MR**	**64 (66.0)**
Rheumatic	33 (34.0)
Degenerative	30 (31.0)
Endocarditis	1(1.0)
**Secondary MR**	**33 (34.0)**
Atrial	7 (7.2)
Ventricular (ischaemic 20, DCM 6)	26 (26.8)
**Regurgitant jet**	
Direction—central / eccentric	22 (22.7) / 75 (77.3)
Number—single / multiple	89 (91.8) / 8 (8.2)
Timing—holosystolic / mid‐late systolic	82 (84.5) / 15 (15.5)

Abbreviation: DCM, dilated cardiomyopathy; MR, mitral regurgitation. Percentages are calculated on the whole cohort (N = 97).

### 3.3 Chamber Remodeling and Severity

Mean LV ejection fraction (LVEF) was 51.7 ± 14%, and 26.8% had impaired LVEF (≤ 40%); LV dimensions were dilated in 47.4% and the left atrium in 90.7%. The right ventricle was dilated in 33% and its systolic function impaired in 18.6% (mean systolic pulmonary artery pressure 42.5 ± 14.6 mmHg). LV dilatation and LVEF impairment were more frequent in functional MR. Overall, 61 patients (62.9%) had severe MR; quantitative and semi‐quantitative parameters were markedly higher in severe than in moderate MR (3D VCA 0.86 ± 0.47 vs. 0.35 ± 0.21 cm^2^; EROA 0.49 ± 0.18 vs. 0.23 ± 0.09 cm^2^; regurgitant volume 61.3 ± 19.0 vs. 37.8 ± 14.8 mL; VC width 6.9 ± 1.7 vs. 4.8 ± 1.2 mm).

### 3.4 Correlations Between 3D VCA and 2D Parameters

**FIGURE 2:**
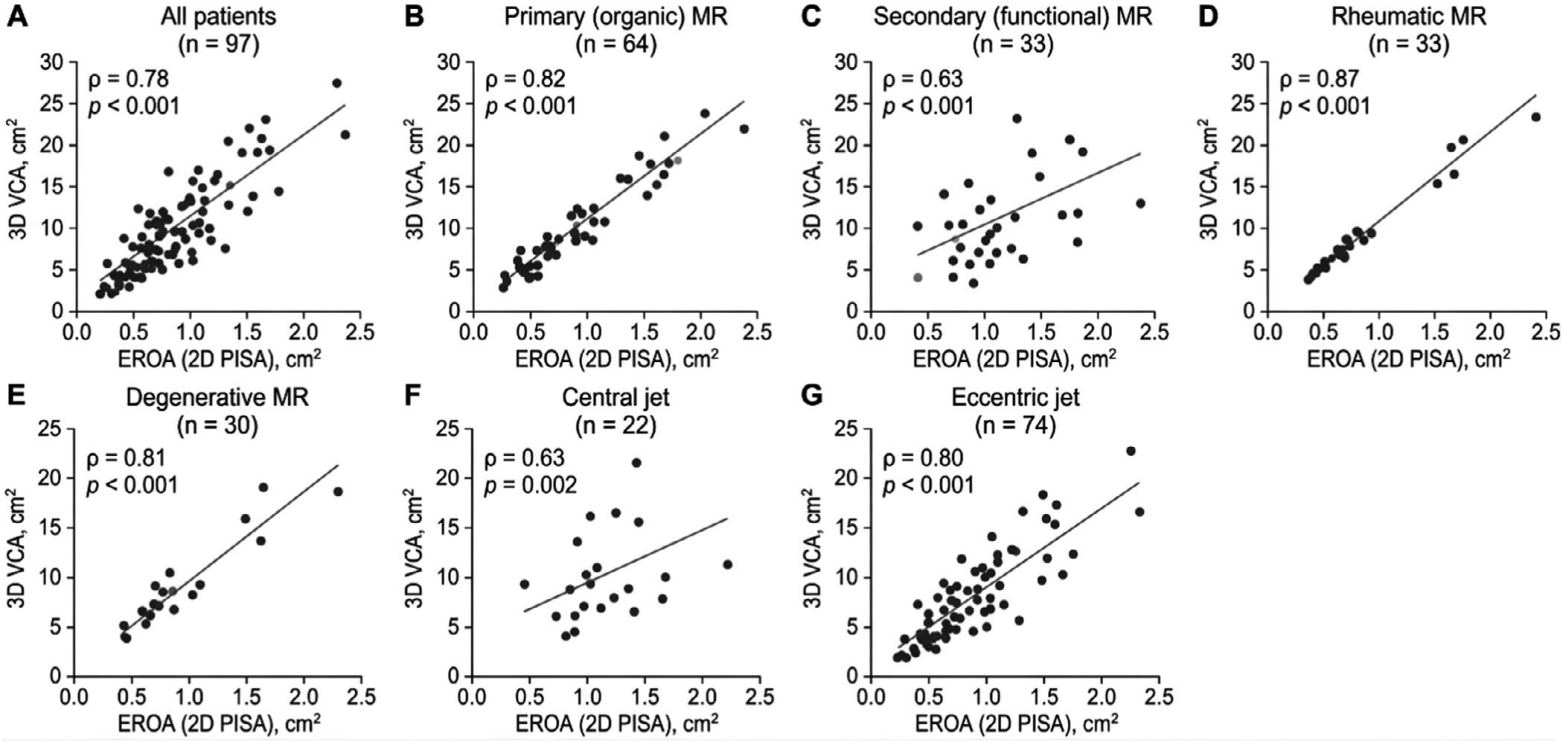
Correlation between 3D vena contracta area and 2D effective regurgitant orifice area (PISA). Correlations are shown for the whole cohort (A), primary MR (B), secondary MR (C), rheumatic MR (D), degenerative MR (E), central jets (F), and eccentric jets (G). Abbreviations EROA, effective regurgitant orifice area; MR, Mitral regurgitation; PISA, proximal isovelocity surface area; VCA, vena contracta area; ρ, Spearman correlation coefficient.

In the whole cohort, 3D VCA correlated positively and strongly with VC width (rho = 0.65, *p* < 0.001) and with PISA‐derived EROA (rho = 0.78, *p* < 0.001). Correlations with EROA were strong to very strong in organic (rho = 0.82), rheumatic (rho = 0.87), degenerative (rho = 0.81) and eccentric (rho = 0.80) MR, and weaker in secondary MR (rho = 0.63); in atrial functional MR the correlation was weak and non‐significant (Figure 2).

### 3.5 Diagnostic Performance and Cut‐offs

**FIGURE 3:**
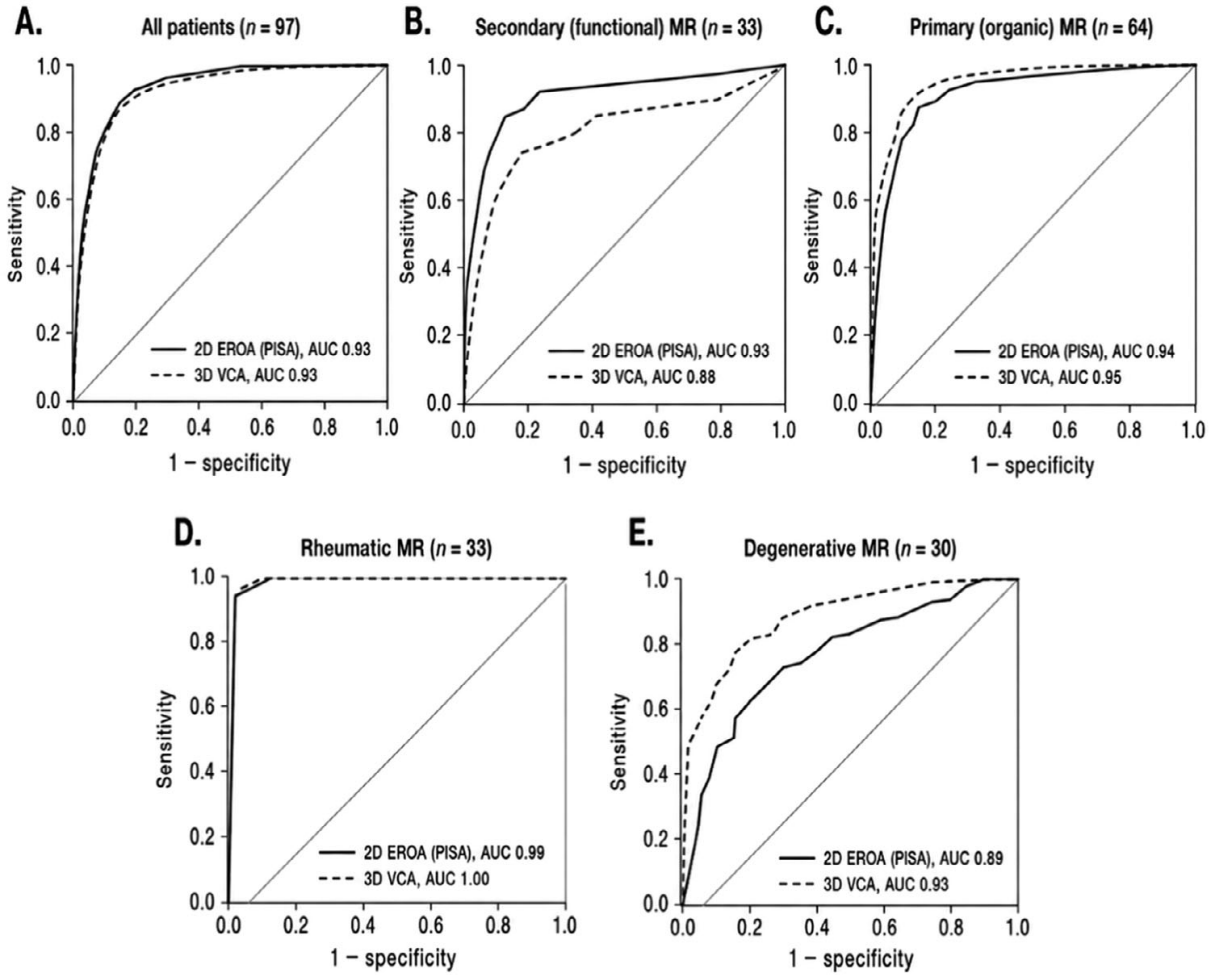
Receiver operating characteristic curves of three‐dimensional vena contracta area and PISA‐derived effective regurgitant orifice area. Curves are shown for the whole cohort (A), secondary MR (B), primary MR (C), rheumatic MR (D), and degenerative MR (E). Abbreviations AUC, area under the curve; EROA, effective regurgitant orifice area; MR, mitral regurgitation; PISA, proximal isovelocity surface area; VCA, vena contracta area.

For severe MR, 3D VCA and EROA showed identical overall discrimination (AUC 0.93). The optimal 3D VCA cut‐off was > 0.43 cm^2^ (sensitivity 96.7%, specificity 86.1%; Youden 0.83) and the EROA cut‐off was > 0.32 cm^2^ (sensitivity 73.8%, specificity 97.2%). Aetiology‐specific 3D VCA cut‐offs were > 0.42 cm^2^ in rheumatic MR (AUC 1.00; sensitivity and specificity 100%), > 0.55 cm^2^ in degenerative MR (AUC 0.93), > 0.43 cm^2^ in primary MR (AUC 0.95; sensitivity 100%) and > 0.39 cm^2^ in secondary MR (AUC 0.88; sensitivity 100%, specificity 75%). ROC characteristics are summarized in Table 4 and the curves are shown in Figure 3.

**TABLE 4:** Receiver operating characteristic analysis of Two‐dimensional effective regurgitant orifice area and three‐dimensional vena contracta area for identifying severe mitral regurgitation in the overall population and according to mitral regurgitation aetiology (*N* = 97). Values are presented as area under the curve with 95% confidence intervals, optimal cut‐off values, sensitivity, specificity, and Youden index.

Subgroup / parameter	*n*	AUC (95% CI)	Cut‐off (cm^2^)	Se (%)	Sp (%)	J
**All patients (*N* = 97)**						
EROA (PISA)	97	0.93 (0.87‐0.99)	> 0.32	73.8	97.2	0.71
3D VCA	97	0.93 (0.86‐0.99)	> 0.43	96.7	86.1	0.83
**Secondary MR (*n* = 33)**						
EROA (PISA)	33	0.93 (0.84‐1.00)	> 0.22	95.2	83.3	0.79
3D VCA	33	0.88 (0.73‐1.00)	> 0.39	100	75.0	0.75
**Primary MR (*n* = 64)**						
EROA (PISA)	64	0.94 (0.87‐1.00)	> 0.35	87.5	95.8	0.83
3D VCA	64	0.95 (0.88‐1.00)	> 0.43	100	87.5	0.88
**Rheumatic MR (*n* = 33)**						
EROA (PISA)	33	0.99 (0.96‐1.00)	> 0.35	89.5	100	0.89
3D VCA	33	1.00 (1.00)	> 0.42	100	100	1.00
**Degenerative MR (*n* = 30)**						
EROA (PISA)	30	0.89 (0.75‐1.00)	> 0.35	85.7	88.9	0.75
3D VCA	30	0.93 (0.82‐1.00)	> 0.55	95.2	77.8	0.73

Abbreviation:AUC: area under the curve; CI: confidence interval; EROA: effective regurgitant orifice area; J: Youden index; MR: mitral regurgitation; PISA: proximal isovelocity surface area; Se: sensitivity; Sp: specificity; 3D VCA: three‐dimensional vena contracta area.

### 3.6 Management and Outcome

All patients with moderate MR were managed medically. Among patients with severe MR, 47.5% underwent surgery (mitral valve replacement 37.7%, repair 10%), 1 case of percutaneous edge‐to‐edge repair and 6.6% cardiac resynchronization therapy. During the study, 13 patients (13.4%) died, all with severe MR (21.3% of the severe group), with no deaths among patients with moderate MR.

## 4 Discussion

In this first Tunisian prospective cohort, 3D VCA was feasible across a diverse range of MR aetiologies and discriminated severe MR with excellent accuracy, matching or exceeding PISA‐derived EROA while avoiding its geometric assumptions. 3D VCA correlated strongly with both 2D vena contracta and EROA, and we derived aetiology‐specific severity cut‐offs.

The aetiological profile reflects an epidemiological transition: degenerative MR was frequent, as in Western series, yet rheumatic disease still accounted for one third of cases—a proportion far higher than in most European and North American cohorts [19, 20]. This is clinically important because rheumatic leaflets, with restricted motion and irregular orifices, are precisely the substrate in which 2D Doppler quantification is least reliable. The very strong VCA‐EROA correlation (rho = 0.87) and a perfect ROC (AUC 1.00; cut‐off 0.42 cm^2^) in rheumatic MR support the feasibility and value of 3D planimetry in this under‐represented group, in line with the limited available evidence on 3D imaging of rheumatic valves [21].

Our overall 3D VCA cut‐off (0.43 cm^2^) is close to the 0.41 cm^2^ reported by Goebel et al. for severe MR and consistent with other 3D series [12, 14, 21, 22]. The higher cut‐off in degenerative MR (0.55 cm^2^) is consistent with the eccentric, often prolapse‐related jets in this group [23], whereas the lower secondary‐MR cut‐off (0.39 cm^2^) accords with guideline thresholds that recognize the prognostic impact of smaller orifices in functional disease [9, 24]. The known tendency of the PISA method to overestimate the orifice in mid‐systolic, dynamic and non‐hemispheric convergence is mitigated by direct 3D planimetry [25, 26].

Because 3D VCA traces the actual orifice, it is particularly advantageous for eccentric, multiple and non‐circular jets, where VC width and PISA are most limited [26], this is reflected in the stronger VCA correlations observed in eccentric MR. In atrial functional MR—where the orifice is crescentic and the jet often dynamic—correlations were weakest [27], underlining that no single parameter is sufficient and that 3D VCA should complement, rather than replace, integrative grading.

Clinically, aetiology‐specific 3D VCA cut‐offs may improve the consistency of severity grading and, ultimately, the timing of intervention, especially in laboratories serving rheumatic‐endemic populations. The findings also support routinely adding a focused 3D color‐Doppler acquisition during TEE in patients with at least moderate MR.

This study has limitations. It was conducted in a single center with a modest sample size, and some aetiological subgroups (notably atrial and endocarditis‐related MR) were small, limiting subgroup precision. The reference standard was an integrative 2D multiparametric assessment rather than an independent gold standard such as cardiac magnetic resonance [13, 28]; the derived cut‐offs therefore require external confirmation. Manual tracing and image quality may affect 3D measurements, and inter‐observer reproducibility was not formally quantified here. Another limitation of our study is that 3D TTE assessment of MR vena contracta was not evaluated. Consequently, direct comparison between 3D TTE and 3D TEE measurements could not be performed.

Multicenter studies in North African and other rheumatic‐endemic settings, ideally with cardiac magnetic resonance adjudication, are needed to validate aetiology‐specific 3D VCA thresholds and to define practical guidance for 3D MR quantification where rheumatic valve disease remains prevalent.

## 5 Conclusion

In this Tunisian cohort with diverse MR aetiologies, 3D VCA was feasible and showed good diagnostic accuracy for grading MR severity. It correlated strongly with 2D quantitative parameters and yielded exploratory aetiology‐specific cut‐offs: 0.43 cm^2^ overall; 0.42 cm^2^ for rheumatic MR, 0.55 cm^2^ for degenerative MR, and 0.39 cm^2^ for secondary MR. These findings suggest that 3D VCA may enhance multiparametric grading, particularly in rheumatic and complex MR, but require validation in larger multicenter studies.

## Funding

This research received no external funding.

## Ethics of Approval Statement

The study was approved by the Ethics Committee of the Faculty of Medicine of Sousse and conducted in accordance with the Declaration of Helsinki.

## Patient Consent Statement

Written informed consent was obtained from all participants prior to inclusion in the study

## Conflicts of Interest

The authors declare no conflicts of interest.

## Use of Generative Artificial Intelligence

Generative AI tools were used to assist with language editing and formatting of the manuscript, under the authors' supervision; the authors reviewed and approved all content and are fully responsible for it.

## Data Availability

The data supporting the findings are available from the corresponding author on reasonable request.
